# Tracking Organophosphates: New Method for Assessing Long-Term Dietary Exposures

**DOI:** 10.1289/ehp.123-A135

**Published:** 2015-05-01

**Authors:** Carol Potera

**Affiliations:** Carol Potera, based in Montana, also writes for *Microbe*, *Genetic Engineering News*, and the *American Journal of Nursing*.

Researchers often use urinary biomonitoring as the basis for estimating exposures to organophosphate pesticides (OPs), including dietary exposures. In this issue of *EHP* investigators report a new method to estimate long-term exposure to OPs via produce.^1^ This method appears to be an improvement over estimates based on urine biomarkers, which reflect exposure only in the previous few days.

The researchers analyzed dietary data for nearly 4,500 men and women enrolled in the Multi-Ethnic Study of Atherosclerosis (MESA), a long-term study of cardiovascular disease risk factors in older people. Every two years participants completed a food frequency questionnaire about their typical consumption of an extensive list of foods, including 20 specific fruits and vegetables. They also were asked whether they ate organically grown produce “seldom or never,” “sometimes,” or “often or always.”

**Figure:**
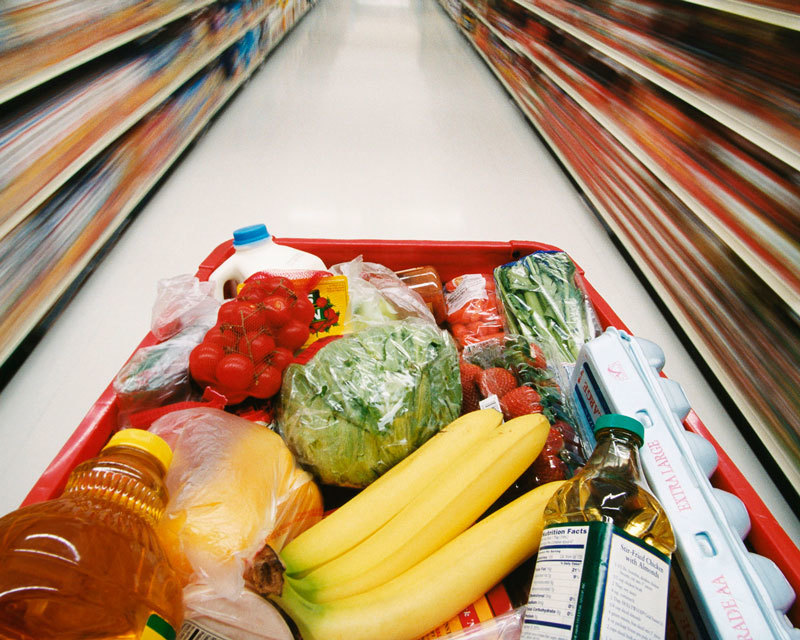
Consumers who want to avoid organophosphates but can’t afford to go completely organic can take a targeted approach, choosing organic only for fruits and vegetables that tend to have the highest pesticide residues. © Steve Craft/Masterfile/Corbis

The authors estimated individuals’ OP exposures by combining this dietary intake information with pesticide residue data reported by the U.S. Department of Agriculture (USDA). For a subset of participants, they then compared estimated individual exposure levels against concentrations of OP metabolites known as dialkylphosphates (DAPs) measured in urine samples.

Participants in this subset who reported often or always eating organic produce had the lowest urinary concentrations of DAP (median 106 nmol/g creatinine), while those who rarely or never ate organic produce had the highest DAP concentrations (median 163 nmol/g creatinine); those who sometimes ate organic produce fell in between (median 121 nmol/g creatinine). Importantly, these comparisons were among people who reported eating comparable amounts of fruits and vegetables. This avoided potential confounding by overall produce intake (in the larger cohort, the authors found that people who reported eating more organic produce also tended to eat more produce overall).^1^

“The health benefits of eating fruits and vegetables—whether organic or conventional—are well established. Our research strengthens claims^2^ that selecting organic produce reduces exposure to OPs,” says study leader Cynthia Curl, now an assistant professor at Boise State University.

Most OPs break down to DAPs, yet specific OPs can vary widely in terms of toxicity.^3^ The USDA’s Pesticide Data Program measures residues of more than 450 pesticides in foods on an “as eaten” basis—for instance, bananas are peeled before they are tested.^4^ By combining food consumption information and USDA residue data for specific pesticides on specific foods, researchers may be able to estimate exposures more accurately in future studies on pesticides and health.

Curl’s paper “breaks important methodological ground by describing a relatively low-cost, noninvasive method for the characterization of long-term dietary exposures,” says Charles Benbrook, program leader of the Measure to Manage program at the Washington State University Center for Sustaining Agriculture and Natural Resources. “Many claims are made today about food quality, and they need intense scrutiny.” Benbrook was not involved in the study.

Curl plans to use the novel technique to examine how pesticide intake affects neurocognitive end points in the MESA population. Exposures to OPs (and other pesticides) have been associated in some studies with increased risk for dementia in adults^5^ and impaired intellectual development in children.^6^

Surveys suggest that about 40% of Americans buy some organic food.^7^ However, the higher cost of organic produce can put an organic diet out of reach for some consumers. “If you want to reduce your OP exposure, but can’t afford to eat exclusively organic, a targeted approach to buy organically may reduce your exposure,” says Curl.

She suggests using the Environmental Working Group’s Dirty Dozen™ and Clean Fifteen™ lists as a guide to deciding when to choose organic produce. The Dirty Dozen list includes fruits and vegetables that tend to have the highest levels of pesticide residues—for 2015 the list includes apples, peaches, and nectarines. The Clean Fifteen, on the other—which includes avocados, pineapples, and corn—are the items that tend to have the lowest pesticide residues.^8^ A new review in *Consumer Reports* gives similar advice.^9^
